# Impact of Angiotensin I-converting Enzyme Inhibitors and Angiotensin II Type-1 Receptor Blockers on Survival of Patients with NSCLC

**DOI:** 10.1038/srep21359

**Published:** 2016-02-17

**Authors:** Lili Miao, Wei Chen, Ling Zhou, Huanying Wan, Beili Gao, Yun Feng

**Affiliations:** 1 Department of Respiration, Ruijin Hospital, School of Medicine, Shanghai Jiao Tong University, Shanghai, China; 2 Department of Respiration, YiZheng People’s Hospital, Jiangsu, China

## Abstract

It has been shown that angiotensin I-converting enzyme inhibitors (ACEIs) and angiotensin II type-1 receptor blockers (ARBs) can decrease tumor growth and tumor-associated angiogenesis and inhibit metastasis. Epidermal growth factor receptor (EGFR) mutations are found in approximately 30% of patients with advanced non-small cell lung cancer (NSCLC) in East Asia and in 10–15% of such patients in Western countries. We retrospectively identified 228 patients with histologically confirmed advanced NSCLC and 73 patients with early stage disease; 103 of these patients took antihypertensive drugs, and 112 received treatment with EGFR tyrosine kinase inhibitors (TKIs). There was a significant difference in progression-free survival after first-line therapy (PFS_1_) between the ACEI/ARB group and the non-ACEI/ARB group. For the patients treated with TKIs, there was a significant difference in PFS but not in overall survival (OS) between the ACEI/ARB group and the non-ACEI/ARB group. For the patients with advanced NSCLC, there was a significant difference in PFS_1_ between the ACEI/ARB group and the non-ACEI/ARB group. ACEI/ARB in combination with standard chemotherapy or TKIs had a positive effect on PFS_1_ or OS, regardless of whether the lung cancer was in the early or advanced stage.

Lung cancer causes 1.4 million deaths per year worldwide. The 5-year survival rate of patients with advanced-stage (inoperable) non-small cell lung cancer (NSCLC) is 18%, with a median survival of 6–12 months. The main treatments for NSCLC are surgery, chemotherapy, targeted therapy and immunotherapy.

Angiotensin I-converting enzyme inhibitors (ACEIs) and angiotensin II type-1 receptor blockers (ARBs) are the most widely used antihypertensive drugs. The renin-angiotensin system (RAS) is involved in the regulation of arterial pressure. Large epidemiological studies have revealed potentially protective effects of RAS against cancer^1^,^2^, although some of the results remain controversial^3^,^4^,^5^,^6^,^7^,^8^. The local RAS reportedly induces angiogenesis and tumor proliferation by promoting vascular endothelial growth factor (VEGF) or epidermal growth factor receptor (EGFR) expression^9^,^10^. Angiotensin II, which is a growth factor, has been shown to stimulate tumor growth^11^,^12^. ACEIs suppress the local RAS by reducing the production of angiotensin II, whereas ARBs selectively block the action of angiotensin II type-1 receptor (AT1R). Previous studies have suggested that ACEIs and ARBs might decrease tumor growth and tumor-associated angiogenesis and inhibit metastasis^13^,^14^. It has been reported that overexpression of angiotensin II-converting enzyme (ACE2) inhibits lung cancer proliferation and angiogenesis^15^. Recent studies reported that the use of ACEIs or ARBs was associated with longer overall survival (OS) in patients with advanced gastric cancer or lung cancer who received combination chemotherapy as first-line treatment^16^. Studies have reported reduced rates of distant metastasis and decreased mortality risk in ACEI or ARB users with prostate, colorectal or breast cancer^17^,^18^,^19^. Approximately 30% of patients with advanced NSCLC in East Asia harbor EGFR mutations. However, there have been no relevant studies of Asian NSCLC patients, particularly those receiving TKIs. We therefore conducted a retrospective study to identify the role of RAS inhibition in the outcomes of patients with NSCLC in China. Moreover, we also examined the influence of ACEIs/ARBs in NSCLC patients receiving TKIs.

## Patients and Methods

### Ethics

The study protocol was approved by the Coordinating Ethics Committee of Ruijin Hospital. We confirmed that informed consent was obtained from all subjects, and the study methods were conducted in accordance with the approved guidelines.

### Patient and clinical data

We retrospectively identified 228 patients with histologically confirmed advanced NSCLC (stage IIIb or IV) and 73 patients with confirmed stage I, II or IIIa disease who presented at our hospital between January 2000 and December 2014 and received at least one cycle of first-line platinum-based chemotherapy. Among these patients, 73 with stage I, II or IIIa disease underwent surgery before chemotherapy. The hospital’s electronic database contains all of the individual results of any laboratory test during in- or outpatient care administered by our hospital, together with detailed data on drugs, the doses and timing of any administered chemotherapy and hospital discharge reports, including complete medication records beyond antineoplastic chemotherapy. Performance status was recorded for each patient. Follow-up data were extracted from the patients’ records. In addition to the use of ACEIs or ARBs, additional administration of β-blockers, calcium antagonists, and other antihypertensive drugs was noted. Overall, 112 patients were treated with TKIs (gefitinib, erlotinib or icotinib), either as initial therapy and in combination with chemotherapy. The characteristics of the patients treated with EGFR-TKIs are shown in supplementary table 1, and those of the other patients are shown in supplementary table 2.

### Statistical analysis

The treatment outcomes were OS and progression-free survival in first-line therapy (PFS_1_). Survival was calculated from the first day of first-line platinum-based chemotherapy until patient death or last visit. The efficacy analysis was based on the intent-to-treat population. OS was defined as the time between the date of diagnosis of recurrent or metastatic disease and the date of death from any cause. Progression-free survival (PFS) was defined as the time from the date of diagnosis of recurrent or metastatic disease to the date of disease progression or death from any cause. The Kaplan-Meier method was employed to estimate the probability of survival, and survival differences were analyzed using the log-rank test. The χ^2^ test or Fisher’s exact test was used to compare categorical variables. All reported p values were the results of two-sided tests, and p < 0.05 was considered statistically significant. Statistical analyses were performed using SPSS, version 19.0 (Chicago, IL, USA).

## Results

### Patient characteristics

Between January 2000 and December 2014, 228 patients with advanced NSCLC were treated with platinum-based first-line chemotherapy in our hospital, and another 73 patients with confirmed stage I, II or IIIa disease underwent surgery before adjuvant chemotherapy. Table 1 shows the characteristics of the ACEI/ARB group (n = 52) and the non-ACEI/ARB group (n = 249). There were 112 patients who received sequential treatment with TKIs. The baseline characteristics were generally the same in the ACEI/ARB and non-ACEI/ARB groups. The characteristics of particular patient subgroups, including advanced NSCLC patients, patients who underwent surgery and EGFR-TKI-treated patients, are shown in supplementary Table 3. In total, 103 patients received medication for hypertension (Table 2). Of these patients, 52 took an ACEI (n = 25) or ARB (n = 27). Other antihypertensive drugs included calcium channel blockers (n = 40), β-blockers (n = 4), and other drugs (n = 7). Combination antihypertensive regimens including more than two drugs were administered to 10 patients. The types and doses of antihypertensive agents were chosen by cardiology specialists at our hospital. All of the patients continued to receive antihypertensive drugs during cancer treatment.

### Influence of ACEIs/ARBs on all patients

**Figure 1:**
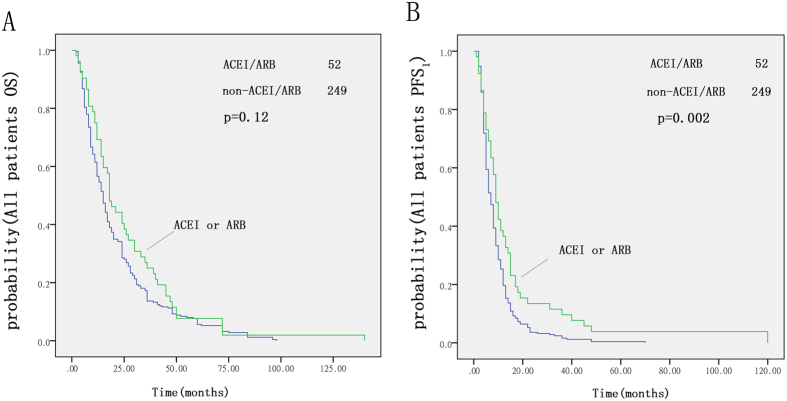
Kaplan-Meier curves for OS (**A**) and PFS_1_ (**B**) with first-line chemotherapy; ACEI/ARB group compared with the non-ACEI/ARB group.

We assessed the possible impact of long-term medication with ACEIs or ARBs on survival. Among all 301 patients, there was a significant difference in PFS_1_ between the ACEI/ARB group and the non-ACEI/ARB group (p = 0.002, Fig. 1B). Recipients of either ACEIs or ARBs had a median PFS_1_ that was 2.4 months longer than that of non-recipients in our Kaplan-Meier analysis (9.2 vs. 6.8 months, p = 0.002, log-rank test). However, there was no significant difference in OS between the ACEI/ARB group and the non-ACEI/ARB group (Fig. 1A).

### Influence of ACEIs/ARBs on patients taking TKIs

**Figure 2:**
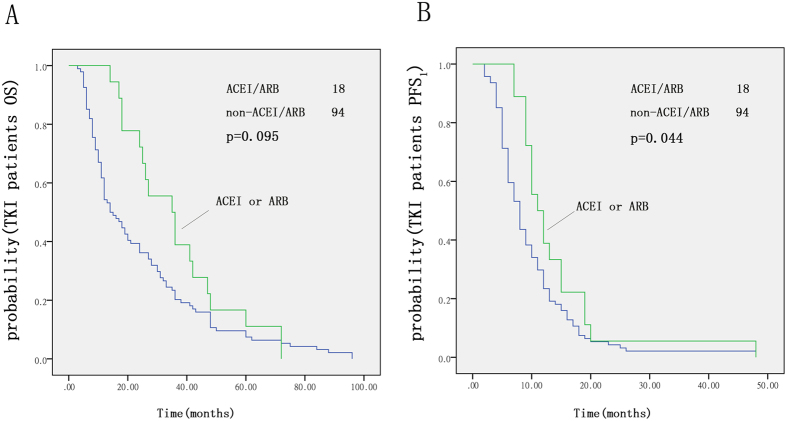
Kaplan-Meier curves for OS (**A**) and PFS (**B**) with TKIs; ACEI/ARB group compared with the non-ACEI/ARB group.

For the 112 patients taking TKIs, there was a significant difference in PFS between the ACEI/ARB group and the non-ACEI/ARB group (p = 0.044, Fig. 2B). Recipients of either ACEIs or ARBs had a median PFS that was 3.2 months longer than that of non-recipients in our Kaplan-Meier analysis (11.2 vs. 8.0 months, p = 0.044, log-rank test). There was no significant difference in OS between the two groups (p = 0.095, Fig. 2A).

### Influence of ACEIs/ARBs on patients who underwent surgery

**Figure 3:**
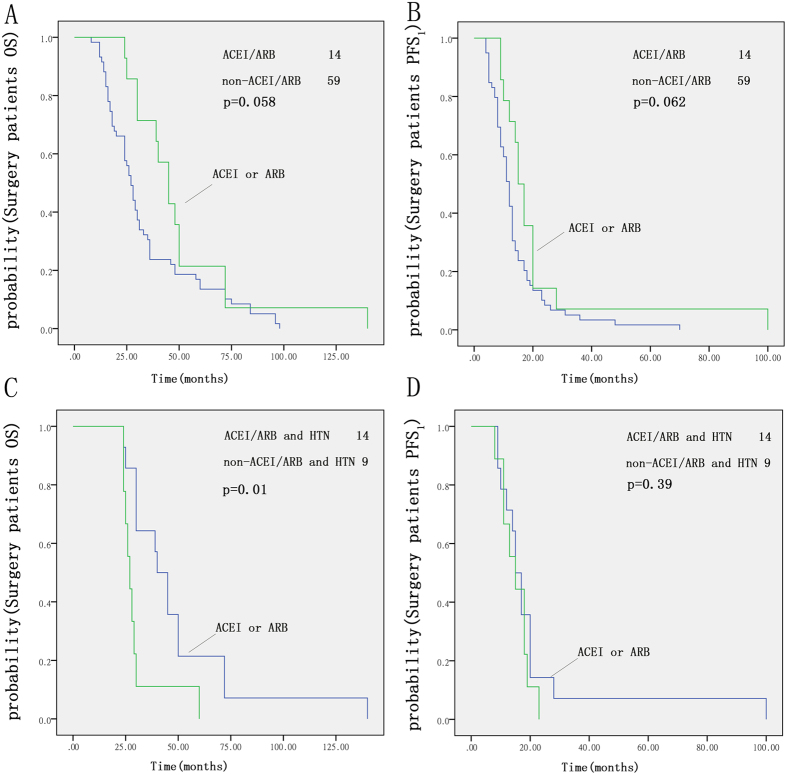
Kaplan-Meier curves for OS (**A**) and PFS_1_ (**B**) in patients who underwent surgery; ACEI/ARB group compared with the non-ACEI/ARB group. Kaplan-Meier curves for OS (**C**) and PFS_1_ (**D**) of patients taking ACEIs/ARBs compared with those taking other antihypertensive drugs.

For the 73 patients with stage I, II or IIIa disease who underwent surgery before chemotherapy, there was no significant difference in OS or PFS between the ACEI/ARB group and the non-ACEI/ARB group (Fig. 3A,B).

There were 23 patients taking antihypertensive drugs. For these 23 patients, there was a significant difference in OS between those taking ACEIs/ARBs and those taking other antihypertensive drugs (p = 0.01, Fig. 3C). There was no significant difference in PFS_1_ between the two groups (Fig. 3D).

### Influence of ACEIs/ARBs on patients with advanced NSCLC

**Figure 4:**
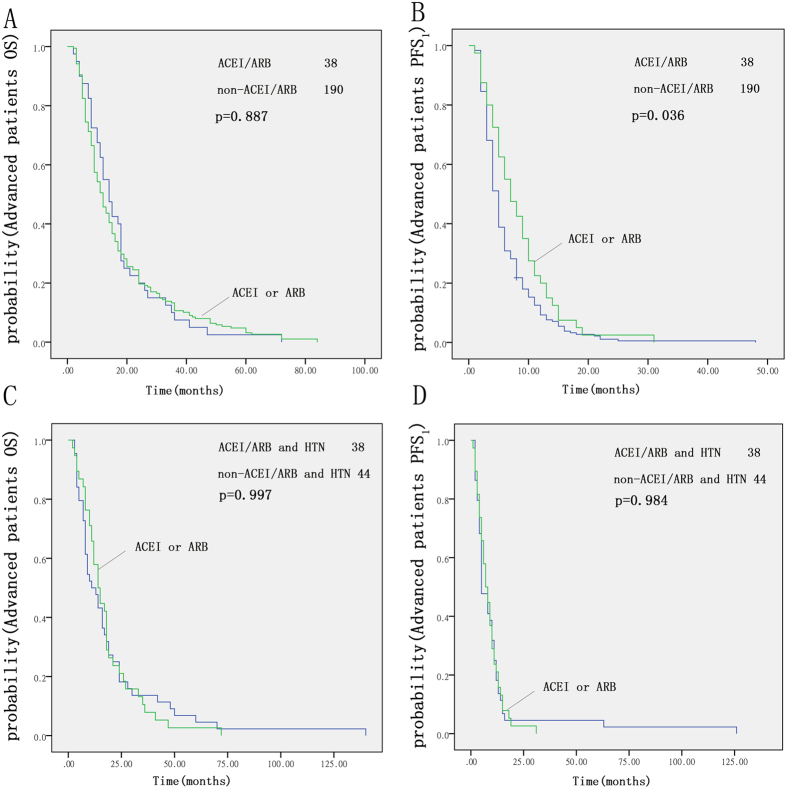
Kaplan-Meier curves for OS (**A**) and PFS_1_ (**B**) of patients with advanced NSCLC; ACEI/ARB group compared with the non-ACEI/ARB group. Kaplan-Meier curves for OS (**C**) and PFS_1_ (**D**) of patients taking ACEIs/ARBs compared with those taking other antihypertensive drugs.

We examined 228 patients with advanced NSCLC who were treated with platinum-based first-line chemotherapy. For these patients, there was a significant difference in PFS_1_ between the ACEI/ARB group and the non-ACEI/ARB group (p = 0.036, Fig. 4B). Recipients of either ACEIs or ARBs had a median PFS_1_ that was 2.1 months longer than that of non-recipients in our Kaplan-Meier analysis (7.3 vs. 5.2 months, p = 0.036, log-rank test). There was no significant difference in OS between the two groups (Fig. 4A).

For the 82 advanced NSCLC patients with hypertension, there was no significant difference in PFS_1_ or OS between those taking ACEIs/ARBs and those taking other antihypertensive drugs (Fig. 4C,D).

## Discussion

This retrospective study is the first analysis to reveal the role of RAS inhibition on the outcomes of NSCLC patients with hypertension who took TKIs. We found that the PFS was 3.2 months longer for these patients than for those in the control group, but OS was not prolonged. Our study also observed, for the first time, the effects of ACEIs or ARBs on an Asian population of patients with advanced NSCLC who underwent surgery. The distribution of established major risk factors (age, sex, histology, stage, and performance status) in both groups was comparable.

Both *in vitro* and *in vivo* studies have shown that ACEIs and ARBs can suppress cell proliferation and tumor metastatic growth in various cancers, including breast cancer, lung cancer, and prostate cancer. Angiogenesis appears to be an important process through which the RAS system exerts pro-tumorigenic effects, and ACEIs and ARBs reduced the expression of VEGF and other angiogenic factors in cell lines^20^ and in animal models^21^. Components of the RAS are expressed at various cancer sites and might contribute to processes that are important for cancer progression, including cell proliferation and apoptosis^22^. The binding of angiotensin II to its type 1 receptor activates the mitogen-activated protein kinase/signal transducer and activator of transcription (MAPK/STAT) pathway, which plays a role in the responses to many growth factors that regulate cell proliferation, differentiation and apoptosis^23^. It has been reported that EGFR is transactivated by AT1R and transinactivated by the type 2 receptor(AT2R). Epidermal growth factor (EGF), among other things, exerts its effects by activating the MAPK/STAT pathway, leading to the development of anti-EGFR drugs as a powerful strategy for anticancer therapy^24^. It has been reported that the activation of similar downstream effectors can directly transactivate EGFR induced by stimulation of AT1R^25^. Our study is the first report that the PFS of NSCLC patients taking TKIs and ACEIs might be prolonged.

In all of the patients studied, there was a significant difference in PFS_1_, but not in OS, between the ACEI/ARB group and the non-ACEI/ARB group. For the advanced NSCLC patients, the use of ACEIs or ARBs was associated with a longer PFS_1_, regardless of whether they had hypertension. However, compared with the use of other hypertension drugs, the use of ACEIs or ARBs did not improve the PFS or OS of advanced NSCLC patients. Our findings revealed that inhibition of the RAS in human NSCLC might improve PFS, but there was little influence on OS. A favorable impact of RAS inhibition on clinical outcomes has also been reported in advanced pancreatic cancer^26^. Previous studies have shown that long-term use of lisinopril is associated with decreased NSCLC metastasis^27^. Two studies reported 44% and 48% reductions in the risk of overall mortality with ACEIs and ARBs, respectively^28^,^29^. In retrospective cohort studies of pancreatic cancer and advanced NSCLC, ACEIs and ARBs were statistically associated with longer patient survival when used with standard first-line chemotherapy^30^. However, we did not find a significant difference in OS for all patients or for the advanced NSCLC patients taking ACEIs/ARBs, although these drugs may prolong PFS_1_. Differences in patient race and the number of patients in our study might have caused discrepant results compared with those of previous studies.

In the patients with hypertension and early NSCLC who underwent surgery, the use of ACEIs or ARBs as opposed to other antihypertensive drugs (calcium channel blockers, β-blockers and other drugs) was associated with a longer OS, although there was no difference in PFS_1_. Similar data have not been previously reported.

This study had some limitations, including the retrospective and non-randomized nature of the review. Furthermore, our study did not consider other important issues, such as toxicity profiles, treatment compliance, or quality of life. The number of patients in our study was limited; therefore, larger-scale research is necessary for further verification.

## Conclusion

Despite the limitations of this study, this is the first report that ACEIs/ARBs increased the PFS of patients with NSCLC who received TKIs. Our clinical observations also revealed that ACEIs/ARBs had positive effects on PFS and OS, regardless of whether the lung cancer was in the early or advanced stage. A prospective study with a larger population is warranted to confirm our hypothesis.

## Additional Information

**How to cite this article**: Miao, L. *et al.* Impact of Angiotensin I-converting Enzyme Inhibitors and Angiotensin II Type-1 Receptor Blockers on Survival of Patients with NSCLC. *Sci. Rep.* **6**, 21359; doi: 10.1038/srep21359 (2016).

## Supplementary Material

Supplementary Information

## Figures and Tables

**Table 1:** Patient characteristics

Characteristic	ACEI/ARB	Non-ACEI/ARB	p value
Age, years			
Median (range)	69 (35–89)		
≤65	17	110	0.13
>65	35	139	
Sex			
Male	35	138	0.07
Female	17	111	
PS			
0	20	100	0.87
≥1	32	149	
Grade			
Good/moderate	23	140	0.07
Poor	29	109	
Chemotherapy			
Only first-line	20	69	0.09
Second-line or more	32	180	

**Table 2:** Number of patients receiving antihypertensive drugs

Antihypertensive agent	Patients, n
ACEIs/ARBs	52
Calcium channel blockers	40
β-blockers	4
Other drugs	7
