# Supplementary material for: Impact of Angiotensin I-converting Enzyme Inhibitors and Angiotensin II Type-1 Receptor Blockers on Survival of Patients with NSCLC
Source: Sci Rep. 2016 Feb 17;6:21359. doi: 10.1038/srep21359 (PMC4756359; doi:10.1038/srep21359)
Supplement: Supplementary Information [file srep21359-s1.pdf]

Impact of Angiotensin I-converting Enzyme Inhibitors and Angiotensin II Type-1 Receptor Blockers on Survival of Patients with NSCLC

Lili Miao[1, 2,*], Wei Chen[1,*], Ling Zhou[1], Huanying Wan[1], Beili Gao[1], Yun Feng[1]

[1]Department of Respiration, Ruijin Hospital, School of Medicine, Shanghai Jiao Tong University, Shanghai, China; [2]Department of Respiration, YiZheng People's Hospital, Jiangsu, China.

Supplementary table 1 EGFR-TKI patients' characteristics

NUMBER	GENDER	AGE (Y)	HISTOLOGY	STAGE	OS (M)	TKI/PFS (M)	TARGETED DRUG	ACEI /ARB
1	F	66	Adenocarcinoma	IV	35.2	14.5	Icotinib	Y
2	M	87	Adenocarcinoma	IIIB	35.8	6.8	Gefitinib	
3	M	78	Squamous cellcarcinoma	IV	15.1	5.2	Icotinib	
4	M	70	Adenocarcinoma	IV	6	4.8	Erlotinib	
5	M	57	Adenocarcinoma	IV	12.9	8.5	Erlotinib	
6	M	66	Adenocarcinoma	IV	18.2	7.2	Erlotinib	Y
7	M	66	Squamous cellcarcinoma	IV	10.2	2.1	Icotinib	
8	F	65	Adenocarcinoma	IV	10.8	7.7	Erlotinib	
9	F	58	Adenocarcinoma	IV	19.2	9	Icotinib	
10	M	73	Squamous cellcarcinoma	IV	10.2	7.9	Erlotinib	
11	F	79	Adenocarcinoma	IV	5.2	4.6	Icotinib	
12	M	73	Adenocarcinoma	IIIB	8.1	6.2	Gefitinib	
13	F	72	Adenocarcinoma	IIIA	20.8	11.1	Erlotinib	
14	F	73	Adenocarcinoma	IIIA	7.9	5.9	Erlotinib	
15	M	66	Adenocarcinoma	IV	10.8	4.9	Erlotinib	
16	F	71	Adenocarcinoma	IV	5.8	5.2	Gefitinib	
17	F	69	Adenocarcinoma	IIIA	27.2	21.7	Gefitinib	
18	F	73	Adenocarcinoma	IV	6	5.8	Gefitinib	
19	M	67	Adenocarcinoma	IV	7.8	6.2	Gefitinib	
20	M	53	Squamous adenocarcinoma	IV	8.2	7.8	Erlotinib	
21	M	65	Adenocarcinoma	IIIA	41.8	15.6	Erlotinib	Y

22	M	71	Non-small cell carcinoma	IIIA	24.1	9.5	Erlotinib	Y
23	M	70	Adenocarcinoma	IIIA	35.8	19.1	Erlotinib	Y
24	M	66	Adenocarcinoma	IIIA	24.9	14.4	Erlotinib	Y
25	M	66	Adenocarcinoma	IV	48.2	11.9	Gefitinib	
26	M	69	Adenocarcinoma	IIIA	47.7	11.9	Gefitinib	
27	F	76	Adenocarcinoma	IIA	88	17.8	Gefitinib	
28	M	67	Adenocarcinoma	IV	18.2	9.8	Gefitinib	
29	F	70	Adenocarcinoma	IV	62	16.2	Gefitinib	
30	F	68	Adenocarcinoma	IV	9.8	8.2	Erlotinib	
31	F	66	Adenocarcinoma	IV	40.4	22.3	Gefitinib	Y
32	F	66	Adenocarcinoma	IV	16.2	6.1	Erlotinib	
33	F	69	Squamous adenocarcinoma	IV	9.2	4.5	Icotinib	
34	F	67	Squamous adenocarcinoma	IV	16.8	4.2	Erlotinib	
35	F	68	Adenocarcinoma	IV	3	4.1	Gefitinib	
36	F	78	Adenocarcinoma	IV	27.8	11.2	Icotinib	
37	F	68	Squamous adenocarcinoma	IV	31	6.8	Icotinib	
38	M	66	Squamous adenocarcinoma	IV	26.2	12.4	Erlotinib	Y
39	M	71	Adenocarcinoma	IV	6.2	3.7	Erlotinib	
40	M	76	Adenosquamous carcinoma	IV	48	5.2	Gefitinib	
41	M	69	Adenocarcinoma	IV	6.8	6.2	Erlotinib	
42	M	77	Adenocarcinoma	IV	17.2	14.5	Gefitinib	Y
43	F	73	Adenocarcinoma	IV	19.8	11.6	Gefitinib	
44	F	28	Non-small cell carcinoma	IV	40.8	6.2	Gefitinib	

45	F	68	Adenocarcinoma	IV	18.8	7.2	Erlotinib	
46	M	79	Adenocarcinoma	IV	36.2	6	Gefitinib	
47	F	71	Adenocarcinoma	IV	8.9	4.9	Gefitinib	
48	M	79	Neuroendocrine carcinoma	IV	5.1	4.1	Erlotinib	
49	F	72	Adenocarcinoma	IV	30.2	8.9	Gefitinib	
50	M	66	Squamous cellcarcinoma	IV	5.9	3.9	Icotinib	
51	M	67	Adenocarcinoma	IV	42.3	15.2	Gefitinib	
52	M	68	Adenocarcinoma	IV	49.7	14.3	Gefitinib	
53	F	76	Adenocarcinoma	IV	11.1	10.8	Gefitinib	
54	F	81	Adenocarcinoma	IV	23.8	12.2	Icotinib	
55	F	69	Adenocarcinoma	IV	13.7	6.8	Gefitinib	
56	M	70	Adenocarcinoma	IV	11.3	5.1	Erlotinib	
57	M	69	Adenocarcinoma	IV	36.3	7.1	Gefitinib	Y
58	F	79	Adenocarcinoma	IV	24.2	15.8	Gefitinib	
59	M	75	Adenocarcinoma	IV	14.2	8.8	Gefitinib	Y
60	M	67	Squamous cellcarcinoma	IV	9.2	4.2	Icotinib	
61	F	74	Adenocarcinoma	IV	8.8	6.8	Erlotinib	
62	F	71	Adenocarcinoma	IV	5.2	4.6	Erlotinib	
63	M	76	Adenocarcinoma	IB	47.7	13	Gefitinib	Y
64	F	77	Adenocarcinoma	IV	11	5.4	Erlotinib	
65	M	67	Adenocarcinoma	IIIB	7.7	3.1	Icotinib	
66	M	68	Adenocarcinoma	IV	4.8	5	Icotinib	
67	M	66	Adenocarcinoma	IV	12.2	6.3	Gefitinib	
68	F	81	Adenocarcinoma	IV	17.8	6.7	Gefitinib	Y

69	F	71	Adenocarcinoma	IV	31	17.9	Gefitinib	
70	F	80	Squamous cellcarcinoma	IV	26.8	19	Erlotinib	
71	F	76	Adenocarcinoma	IV	84	48	Gefitinib	
72	F	67	Adenocarcinoma	IV	43	4.1	Gefitinib	
73	M	66	Adenocarcinoma	IV	27.2	9.5	Gefitinib	Y
74	F	68	Adenocarcinoma	IV	71.7	16.7	Gefitinib	Y
75	M	70	Adenocarcinoma	IV	11.8	7.6	Gefitinib	
76	F	68	Adenocarcinoma	IV	32	15.3	Gefitinib	
77	M	73	Squamous cellcarcinoma	IV	6.6.3	4.3	Erlotinib	
78	M	68	Adenocarcinoma	IV	13.7	10.3	Erlotinib	
79	F	72	Adenocarcinoma	IV	11.8	8.2	Gefitinib	
80	M	89	Adenocarcinoma	IIIB	60	15.2	Gefitinib	
81	M	71	Adenocarcinoma	IV	10.2	1.8	Icotinib	
82	F	80	Squamous cellcarcinoma	IV	5.4	3.3	Erlotinib	
83	M	82	Adenocarcinoma	IV	9.3	8.8	Gefitinib	
84	F	70	Adenocarcinoma	IV	23.8	21.8	Gefitinib	
85	F	69	Adenocarcinoma	IV	12.3	3.9	Erlotinib	
86	F	67	Non-small cell carcinoma	IV	20.2	2.2	Gefitinib	
87	F	70	Adenocarcinoma	IIIA	38.2	18.2	Gefitinib	
88	M	66	Adenocarcinoma	IIIA	95.4	23.2	Gefitinib	
89	M	66	Adenocarcinoma	IIIB	47.2	17.3	Erlotinib	Y
90	F	68	Adenocarcinoma	IB	60.2	18.9	Gefitinib	
91	F	66	Adenocarcinoma	IIIA	29.8	6.2	Gefitinib	
92	F	69	Adenocarcinoma	IV	71.8	9.9	Gefitinib	

93	F	66	Adenocarcinoma	IV	48	12.2	Gefitinib	
94	F	68	Adenocarcinoma	IIIA	28	5.1	Gefitinib	
95	M	63	Adenocarcinoma	IIA	72.2	48	Erlotinib	Y
96	M	75	Adenocarcinoma	IV	33.5	11.5	Erlotinib	
97	M	70	Adenocarcinoma	IIA	34.5	9.7	Erlotinib	
98	M	66	Adenocarcinoma	IIIA	96	11.2	Gefitinib	
99	F	66	Adenocarcinoma	IIIB	74.4	23.4	Gefitinib	
100	F	68	Adenocarcinoma	IIIB	33.5	25.9	Erlotinib	
101	F	80	Adenocarcinoma	IIA	60.6	9.2	Erlotinib	Y
102	M	70	Adenocarcinoma	IIA	35.5	7.8	Erlotinib	
103	M	66	Adenocarcinoma	IIIB	12	7.8	Erlotinib	
104	F	66	Adenocarcinoma	IV	7.2	6.2	Gefitinib	
105	M	70	Adenocarcinoma	IV	7.8	7.5	Gefitinib	
106	M	61	Adenocarcinoma	IV	17	13.1	Erlotinib	
107	F	55	Adenocarcinoma	IIIB	10.1	8.8	Erlotinib	
108	M	53	Adenocarcinoma	IIIA	12.9	12	Erlotinib	
109	M	68	Adenocarcinoma	IIIA	13.6	8.8	Erlotinib	
110	M	45	Adenocarcinoma	IV	8.5	6.5	Erlotinib	
111	M	70	Adenocarcinoma	IV	13.4	7.5	Gefitinib	
112	M	72	Adenocarcinoma	IIIA	9.5	8.2	Gefitinib	

Supplementary table 2 Non-EGFR-TKI patients' characteristics

NUMBER	GENDER	AGE (Y)	HISTOLOGY	STAGE	OS（M）	PFS_1 (M)	Chemo therapy (PFS_1)	ACEI /ARB
1	F	66	Adenocarcinoma	IV	7.5	3.5	AC	Y
2	M	65	Non small cell carcinoma	IV	21.8	6.2	GP	
3	M	50	Squamous cellcarcinoma	IV	20.2	4.2	GP	
4	M	60	Adenocarcinoma	IV	10.3	3.6	GP	
5	M	62	Aenocarcinomad	IIIB	18	4.8	GP	
6	M	62	Adenocarcinoma	IIIA	6.8	1.3	GP	
7	M	47	Adenocarcinoma	IV	5.5	4.1	AC	
8	M	73	Squamous cellcarcinoma	IIIA	4.8	3.7	GC	
9	M	66	Squamous cellcarcinoma	IV	7.8	4.9	TC	Y
10	M	53	Squamous cellcarcinoma	IV	2.8	1.7	GP	
11	M	76	Adenocarcinoma	IV	8.8	4.2	GP	
12	M	68	Squamous cellcarcinoma	IV	4.7	2.2	GP	
13	M	66	Adenocarcinoma	IIIB	19.2	2.2	TP	Y
14	M	59	Adenocarcinoma	IV	9.1	5.1	GC	
15	M	57	Adenocarcinoma	IV	6.2	4.8	AP	
16	M	82	Big cell carcinoma	IV	71.8	9.5	GC	
17	M	51	Adenocarcinoma	IV	6.8	5.1	GP	
18	M	64	Adenocarcinoma	IIIA	6.1	4.8	AP	
19	M	49	Adenocarcinoma	IIIB	5.1	2.8	AP	
20	M	77	Squamous cellcarcinoma	IIIB	7.7	4.1	GP	Y

21	M	67	Squamous cellcarcinoma	IV	9.1	3.2	GC	
22	M	65	Adenocarcinoma	IV	19.5	9.5	GC	
23	M	66	Squamous cellcarcinoma	IIB	140	120	GP	Y
24	M	51	Squamous cellcarcinoma	IV	13.1	3.1	GC	
25	M	60	Squamous cellcarcinoma	IV	3.2	2.9	GC	
26	F	65	Adenocarcinoma	IV	7.2	2.2	GC	
27	M	75	Adenocarcinoma	IV	9.7	2.9	GC	
28	M	72	Adenocarcinoma	IV	12.8	1.9	GP	
29	M	53	Adenocarcinoma	IIIA	12.7	4.8	GC	
30	M	75	Adenocarcinoma	IV	11.5	4.2	GC	
31	M	63	Adenocarcinoma	IV	15.8	6.1	GP	
32	M	77	Squamous cellcarcinoma	IIIA	11.9	8.1	GC	
33	M	60	Adenocarcinoma	IV	17.3	1.9	AP	
34	F	70	Adenocarcinoma	IV	17.2	6.2	GP	
35	F	68	Adenocarcinoma	IV	8.8	4.2	GP	
36	F	65	Adenocarcinoma	IV	11.2	3.8	AC	
37	F	68	Non small cell carcinoma	IV	8.8	5.7	TC	
38	M	71	Adenocarcinoma	IV	15.2	4.8	AP	
39	M	50	Adenocarcinoma	IV	11.8	5.2	PC	
40	M	63	Adenocarcinoma	IIIB	9.2	2.1	GP	
41	M	61	Squamous cellcarcinoma	IV	15.5	7.9	GP	
42	M	66	Non small cell carcinoma	IV	14.8	11.3	GP	Y
43	M	73	Squamous cellcarcinoma	IIIA	5.8	3.2	GP	

44	F	58	Squamous cellcarcinoma	IV	14.5	5.1	GP	
45	M	82	Adenocarcinoma	IV	18.5	17.5	AC	Y
46	M	75	Adenocarcinoma	IV	17.5	10.2	AC	Y
47	M	53	Adenocarcinoma	IV	36.2	2.8	TC	
48	M	57	Adenocarcinoma	IV	23.6	2.1	GC	
49	M	55	Squamous cellcarcinoma	IV	23.9	3.2	GP	
50	M	45	Adenocarcinoma	IV	4.8	1.2	AP	
51	M	59	Adenocarcinoma	IV	28.5	9.2	GP	
52	M	57	Neuroendocrine carcinoma	IV	60.1	4.9	EC	
53	F	54	Adenocarcinoma	IV	4.2	2.7	AP	
54	M	60	Squamous cellcarcinoma	IIIA	16.2	9.8	GP	
55	M	61	Squamous cellcarcinoma	IIIB	3.9	1.8	GP	Y
56	M	76	Adenocarcinoma	IV	4.9	1.8	GP	
57	M	61	Squamous cellcarcinoma	IIB	15.8	5.1	GP	
58	M	73	Squamous cellcarcinoma	IV	3.1	2.1	GP	
59	M	59	Adenocarcinoma	IV	12.2	3.8	AP	
60	M	58	Squamous cellcarcinoma	IV	5.1	4.1	GP	
61	M	61	Squamous cellcarcinoma	IV	2.1	1.3	GP	Y
62	M	68	Adenocarcinoma	IV	15.8	5.2	AP	
63	M	60	Adenocarcinoma	IV	40.9	8.1	GP	
64	M	57	Non small cell carcinoma	IV	51.8	4.7	EC	
65	M	67	Adenocarcinoma	IV	15.3	4.1	GP	
66	F	75	Adenocarcinoma	IV	14.7	2.8	GP	

67	M	70	Squamous cellcarcinoma	IV	3.3	2.1	GP	
68	M	46	Squamous cellcarcinoma	IV	2.5	1.8	GC	
69	F	64	Adenocarcinoma	IV	9.2	3.3	GP	
70	F	63	Adenocarcinoma	IV	20.6	5.2	GP	Y
71	M	41	Squamous cellcarcinoma	IV	6.4	3.1	GP	
72	M	56	Adenocarcinoma	IV	8.8	3.3	GC	
73	M	64	Adenocarcinoma	IIIA	4.2	2.7	AC	
74	M	76	Squamous cellcarcinoma	IIIB	7.2	3.6	GP	Y
75	M	76	Neuroendocrine carcinoma	IIB	8.2	4.9	GC	
76	M	69	Squamous cellcarcinoma	IV	7.8	1.2	GP	
77	M	55	Squamous cellcarcinoma	IV	20.1	2.7	GP	
78	M	74	Adenocarcinoma	IV	12.5	8.8	GC	
79	M	55	Adenocarcinoma	IV	12.2	11.3	GP	Y
80	M	75	Adenocarcinoma	IV	2.5	2.2	GP	
81	M	63	Adenocarcinoma	IV	13.5	10.2	AC	
82	M	54	Adenocarcinoma	IV	7.6	3.8	GP	
83	M	60	Adenocarcinoma	IV	36	4.2	GP	
84	M	67	Adenocarcinoma	IV	14.4	4.3	GP	
85	M	44	Adenocarcinoma	IV	23.9	3.4	GP	
86	F	67	Squamous cellcarcinoma	IV	16.6	2.3	TP	
87	M	76	Adenocarcinoma	IV	5.8	4.2	GP	
88	M	63	Squamous cellcarcinoma	IIIB	8.2	2.2	GP	
89	M	63	Adenocarcinoma	IV	24	4.8	AP	

90	F	72	Adenocarcinoma	IV	13.6	1.5	GC	
91	M	62	Adenocarcinoma	IV	8.2	2.1	GP	
92	M	51	Adenocarcinoma	IV	12.2	6.5	AC	Y
93	M	74	Adenocarcinoma	IV	12.4	10.8	AC	
94	F	52	Adenocarcinoma	IV	7.8	5.5	GP	Y
95	F	65	Adenocarcinoma	IV	35.8	2.2	GP	
96	F	75	Adenocarcinoma	IV	24	8.2	NP	Y
97	M	57	Adenocarcinoma	IV	4.2	3.3	AP	Y
98	F	76	Adenocarcinoma	IV	4.2	3.8	AP	
99	M	62	Squamous cellcarcinoma	IV	4.3	3.7	GC	
100	F	53	Adenocarcinoma	IV	5.5	4.2	TC	
101	M	59	Adenocarcinoma	IV	28.2	4.3	GP	
102	M	74	Adenocarcinoma	IV	14.3	7.9	GC	Y
103	F	75	Adenocarcinoma	IV	8.8	4.9	GP	
104	F	54	Squamous cellcarcinoma	IV	3.2	2.7	GP	
105	F	79	Adenocarcinoma	IV	13.7	1.7	GP	Y
106	F	47	Adenocarcinoma	IV	15.8	3.2	GP	
107	F	58	Adenocarcinoma	IV	8.9	4.7	AP	
108	M	76	Squamous cellcarcinoma	IIIB	6.9	3.7	GP	
109	M	62	Adenocarcinoma	IV	4.8	3.8	AC	
110	F	53	Adenocarcinoma	IV	11.6	3.8	AC	
111	F	55	Adenocarcinoma	IV	3.3	2.2	GP	
112	F	61	Adenocarcinoma	IV	6	5.2	AP	Y
113	M	72	Adenocarcinoma	IV	3.2	2.8	AP	

114	F	52	Adenocarcinoma	IV	6.1	1.6	GC	
115	F	68	Adenocarcinoma	IV	14.4	3.5	GP	
116	F	48	Adenocarcinoma	IV	6.9	3.3	GP	
117	F	51	Adenocarcinoma	IV	5.9	3.2	AC	
118	F	79	Squamous cellcarcinoma	IV	11.2	8.1	NP	Y
119	F	45	Adenocarcinoma	IV	4.5	1.4	AC	
120	F	57	Adenocarcinoma	IV	9.2	4.5	NP	
121	M	61	Squamous cellcarcinoma	IV	5.2	2.7	GP	
122	M	63	Adenocarcinoma	IV	24.5	5.5	AP	
123	M	57	Adenocarcinoma	IV	8.9	4.5	GP	
124	M	57	Adenocarcinoma	IV	5.8	3.1	GP	
125	F	53	Adenocarcinoma	IV	4.2	1.8	TP	
126	M	46	Adenocarcinoma	IV	3.1	1.8	AC	
127	M	59	Adenocarcinoma	IV	6.2	3.1	GP	
128	M	44	Adenocarcinoma	IV	6.8	4.2	AC	
129	M	72	Adenocarcinoma	IV	4.8	2.9	TP	
130	M	66	Adenocarcinoma	IV	7.8	3.3	NP	
131	M	71	Adenocarcinoma	IV	6.2	2.9	NP	
132	M	72	Adenocarcinoma	IV	4.9	2.8	NP	
133	F	62	Adenocarcinoma	IV	4.3	3.2	NP	Y
134	M	68	Squamous cellcarcinoma	IIA	59.8	40	GP	Y
135	M	60	Squamous cellcarcinoma	IIIA	48.1	9.9	GP	
136	M	71	Adenocarcinoma	IIB	12.2	6.5	GP	
137	M	63	Squamous cellcarcinoma	IIIB	10.8	3.8	GP	Y

138	M	61	Squamous cellcarcinoma	IIIA	25.5	7.5	TC	
139	M	60	Adenocarcinoma	IB	60	30.7	AC	
140	M	59	Squamous cellcarcinoma	IIA	17.6	7.9	GP	
141	M	54	Adenosquamous carcinoma	IV	15.2	9.2	GP	Y
142	M	59	Squamous cellcarcinoma	IIIB	17.8	4.4	GP	Y
143	M	49	Adenocarcinoma	IIA	17.2	6.8	GP	
144	F	66	Squamous cellcarcinoma	IB	28.2	8.3	GP	
145	F	71	Squamous cellcarcinoma	IIIB	18.5	10.8	GP	
146	M	57	Squamous cellcarcinoma	IIIA	4.2	1.8	TP	
147	M	63	Adenocarcinoma	IIIA	15.3	9.2	GP	
148	M	61	Squamous cellcarcinoma	IB	120	120	GP	Y
149	F	56	Adenocarcinoma	IB	72	35.6	AC	Y
150	F	70	Adenocarcinoma	IB	50.7	44.7	GP	Y
151	F	70	Adenocarcinoma	IB	45.3	14.5	AP	Y
152	F	57	Adenocarcinoma	IB	25.1	8.8	AC	
153	F	64	Adenocarcinoma	IIB	28.7	6.8	NP	
154	F	65	Adenocarcinoma	IIIA	12.2	3.8	GP	
155	M	65	Adenocarcinoma	IV	11.5	10.4	NP	Y
156	M	51	Adenocarcinoma	IA	55.3	8.2	AP	
157	F	57	Adenocarcinoma	IIIB	39.5	8.3	TP	
158	M	68	Squamous cellcarcinoma	IIA	22.5	4.5	GP	
159	M	54	Adenocarcinoma	IV	29.7	7.7	NP	
160	M	56	Large cell lung carcinoma	IIIA	20.5	5.5	GC	

161	F	78	Adenocarcinoma	IV	33	31	AC	Y
162	F	68	Squamous cellcarcinoma	IV	30.5	4.5	GP	
163	F	56	Adenocarcinoma	IV	71.9	5.5	GC	
164	M	61	Squamous cellcarcinoma	IV	9.2	7.2	GP	
165	M	67	Adenocarcinoma	IB	48.2	12.6	AP	Y
166	M	68	Adenocarcinoma	IIIA	83.8	70	GP	
167	M	67	Squamous cellcarcinoma	IIIB	13.2	10.8	GP	
168	M	77	Squamous cellcarcinoma	IIIB	14.8	12.3	GC	
169	M	42	Adenocarcinoma	IIB	18.5	11.2	GP	
170	M	46	Adenocarcinoma	IB	23.8	12.2	AP	
171	F	59	Adenocarcinoma	IIB	15.3	12.5	AP	
172	M	69	Squamous cellcarcinoma	IV	84.3	11.8	GC	
173	M	43	Adenocarcinoma	IIA	35.5	11.7	AP	
174	M	49	Large cell lung carcinoma	IIIB	17.2	13.3	AC	
175	F	66	Adenocarcinoma	IV	8.8	2.2	AP	
176	F	77	Adenocarcinoma	IIIA	14.4	12.8	GP	
177	F	71	Adenocarcinoma	IB	29.7	11.2	GP	
178	F	61	Adenocarcinoma	IIIB	14.6	12.1	GP	
179	M	66	Adenocarcinoma	IIB	18.2	12.1	GP	
180	M	69	Squamous cellcarcinoma	IIIA	18.1	11.8	GP	
181	M	69	Squamous cellcarcinoma	IIB	24.2	11.2	GP	
182	M	58	Adenocarcinoma	IB	36.5	13.5	GP	
183	M	59	Squamous cellcarcinoma	IIIA	16.9	13.5	GP	

184	M	67	Adenocarcinoma	IIA	29.3	3.8	NP	
185	F	68	Adenocarcinoma	IIA	36	8	GP	
186	M	56	Adenocarcinoma	IB	15.5	9	GP	Y
187	M	58	Adenocarcinoma	IB	45.9	3.1	TP	
188	F	65	Squamous cellcarcinoma	IB	72.4	4.1	GC	
189	M	77	Squamous cellcarcinoma	IB	71.7	36.3	AC	

Supplementary table 3 Subgroup patients' characteristics

Characteristics	ACEI/ARB	Non-ACEI/ARB	p value	HBP	Non-HBP	p value
Subgroup			Advanced NSCLC			
Age, years						
≤65	11	85	0.07	32	70	0.19
>65	27	105		50	76	
Sex						
Male	20	90	0.55	55	96	0.84
Female	18	100		27	50	
PS						
0	19	89	0.72	30	60	0.50
≥1	19	101		52	86	
Grade						
Good/moderate	22	102	0.63	39	80	0.93
Poor	16	88		43	86	
Chemotherapy						
Only first-line	8	70	0.06	40	54	0.08
Second-line or more	30	120		42	92	
Subgroup			Undergoing surgeries			
Characteristics	ACEI/ARB	Non-ACEI/ARB	p value	HBP	Non-HBP	p value
Age, years						
≤65	7	39	0.26	9	12	0.15
>65	7	20		14	40	
Sex						
Male	10	40	0.79	13	30	0.92
Female	4	19		10	22	
PS						
0	9	30	0.37	16	32	0.50

≥1	5	29		7	20	
Grade						
Good/moderate	11	42	0.58	10	24	0.83
Poor	3	17		13	28	
Chemotherapy						
Only first-line	6	27	0.84	8	14	0.49
Second-line or more	8	32		15	38	

Subgroup	EGFR-TKI		
Characteristics	ACEI/ARB	Non-ACEI/ARB	p value
Age, years			
≤65	10	48	0.72
>65	8	46	
Sex			
Male	11	50	0.53
Female	7	44	
PS			
0	12	70	0.49
≥1	6	24	
Grade			
Good/moderate	14	72	0.91
Poor	4	22	
Chemotherapy			
Only first-line	8	22	0.07
Second-line or more	10	72	
